# An Examination of Parent-Reported Facilitators and Barriers to Organized Physical Activity Engagement for Youth With Neurodevelopmental Disorders, Physical, and Medical Conditions

**DOI:** 10.3389/fpsyg.2020.568723

**Published:** 2020-09-29

**Authors:** Nicole V. Papadopoulos, Moira Whelan, Helen Skouteris, Katrina Williams, Jennifer McGinley, Sophy T. F. Shih, Chloe Emonson, Simon A. Moss, Carmel Sivaratnam, Andrew J. O. Whitehouse, Nicole J. Rinehart

**Affiliations:** ^1^Deakin Child Study Centre, School of Psychology, Faculty of Health, Deakin University, Geelong, VIC, Australia; ^2^Monash Centre for Health Research and Implementation, School of Public Health and Preventative Medicine, Monash University, Clayton, VIC, Australia; ^3^Clinical Sciences, Murdoch Children’s Research Institute, Parkville, VIC, Australia; ^4^Department of Paediatrics, University of Melbourne, Parkville, VIC, Australia; ^5^Department of Paediatrics, Monash University, Clayton, VIC, Australia; ^6^Department of Physiotherapy, University of Melbourne, Parkville, VIC, Australia; ^7^Deakin Health Economics, School of Health and Social Development, Faculty of Health, Deakin University, Geelong, VIC, Australia; ^8^Surveillance, Evaluation and Research Program, The Kirby Institute, University of New South Wales, Sydney, NSW, Australia; ^9^Research and Research Training, Charles Darwin University, Palmerston City, NT, Australia; ^10^Telethon Kids Institute, University of Western Australia, Nedlands, WA, Australia

**Keywords:** organized physical activity, positive youth development, disability, facilitators, barriers

## Abstract

Organized physical activity (OPA) is an important contributor to physical, social, and emotional health and well-being; however, young people with disabilities are participating at lower rates than their peers without disabilities. This study aimed to (1) compare facilitators and barriers to OPA for young people with disabilities who currently do and do not participate in OPA and (2) to assess whether groups differed in the type of internal and external assets they reported. Parents of 218 young people (41% with a primary diagnosis of autism spectrum disorder) with a diverse representation of disabilities completed an online survey. Young people were categorized as either participants in OPA (*n* = 131) or non-participants (*n* = 87) by parent report. Non-participation was significantly predicted by the barrier “there are no activities my child enjoys” and by a lack of children’s motivation and happiness during OPA. Significant internal assets differentiating participants from non-participants were the ability to understand simple instructions, love of sport, and meeting physical activity guidelines. Significant external assets were parent and sibling participation in OPA, school type, and household income. The findings from this study have important implications for the design of public health interventions that aim to promote OPA in young people with disabilities, highlighting the need to make activities enjoyable, promote participation of siblings and parents, and support low-income families to participate.

## Introduction

The benefits of regular participation in physical activity (PA) for physical, social, and emotional health and well-being are well-recognized (Janssen and Leblanc, 2010; Ahn and Fedewa, 2011; Moeijes et al., 2018, 2019). One type of PA that children and young people engage in regularly for health and fun is organized physical activity (OPA). OPA is defined as PA organized by a club, association, or other type of organization. It usually comprises training sessions or classes, competitions, or matches supervised or coached by an adult (Australian Sports Commission, 2018; Wiium and Safvenbom, 2019). OPA is an important context in which positive youth development occurs, providing opportunities for social engagement, promoting social skills (Howie et al., 2010), enhancing mental health, and improved quality of life (Cutt et al., 2007; Eime et al., 2013; Moeijes et al., 2019).

Organized physical activity has the potential to provide additional benefits for young people with disability such as promoting inclusion, providing social connection, reducing complications of immobility, enhancing social and emotional well-being, and controlling or slowing functional decline (Murphy and Carbone, 2008; Rosewater, 2009; Anderson and Heyne, 2010; Howells et al., 2019). Yet, despite the plethora of benefits, children and young people with a disability are less likely to engage in OPA than those without a disability (Solish et al., 2010; Shields and Synnot, 2016). To understand the low rates of participation, studies have examined facilitators and barriers to PA in children and young people with a disability (Shields et al., 2012; Martin, 2013); however, there is little research focused explicitly on OPA. This is an important gap as there is evidence to suggest that different personal and environmental factors are associated with OPA participation compared to unorganized (or self-organized) PA (Smith et al., 2010; Noonan et al., 2017; Wiium and Safvenbom, 2019). Furthermore, participation rates are lower for OPA than for unorganized PA in children with a disability (Solish et al., 2010; Arim et al., 2012).

Several theoretical frameworks have been used to understand PA participation of children with disabilities (Ross et al., 2016). They include the theory of Planned Behavior (Ajzen, 1991), self-determination theory (Deci and Ryan, 2000), and socio-ecological frameworks (e.g., Bronfenbrenner, 1979; see Rhodes et al., 2019 for a review of these theoretical frameworks). To address conceptual and terminological inconsistencies relating to the participation construct, the Family of Participation-Related Constructs (fPRC) framework was proposed (Imms et al., 2016, 2017). Participation was considered to include attendance and involvement, the latter being viewed as the experience of participation during attendance, which encompassed motivation, persistence, engagement, social connection, and affect (Imms et al., 2016). Three concepts related to participation and incorporated into the framework were preferences (activities that hold meaning or are valued), sense of self (confidence, satisfaction, self-esteem, and self-determination), and activity competence (capability capacity, performance). The relationships between these within-person factors and participation were hypothesized to be bi-directional; influenced by past participation experiences and, in turn, influencing future participation.

While no theory or combination of theories has sufficiently explained all the variables associated with PA (Bauman et al., 2002), the integration of components from various theories into a multilevel framework is thought to offer the best way to understand and intervene in PA behavior (King et al., 2009; Bauman et al., 2012). One such framework which integrates multiple theories across multiple levels is the positive youth developmental (PYD) framework, a strengths-based interdisciplinary approach that links the young person’s developmental strengths and inherent capacity to thrive with ecological contexts (relationships, resources, communities, opportunities) (Benson et al., 2007). In the PYD framework, OPA is considered to be a developmental context in which PYD can be promoted (Zarrett et al., 2008). It is noted in the PYD literature, however, that OPA does not automatically result in positive outcomes. Aligning with the fPRC framework which distinguishes between attendance and involvement, proponents of PYD suggest that positive outcomes are contingent on the way that OPA is delivered and experienced by the young person (Petitpas et al., 2005). Specifically, the activity needs to be intrinsically rewarding, provide opportunities to learn or acquire life skills (internal assets) such as problem solving and decision-making, and be supported by external assets such as caring adult mentors (coaches), strong peer relationships, parental involvement, and a sense of belonging to the wider community (Holt et al., 2017). The PYD framework fits well within the disability context because of its emphasis on strengths rather than deficit; it positions the young person with a disability as having the same inherent potential to grow and develop as any other young person if they receive support, empowerment, and engagement through positive relationships, contexts, and ecologies (Benson et al., 2007).

While OPA participation is recognized as an important context in which to promote PYD (Holt et al., 2017), many barriers were identified for young people with disabilities in a systematic review of facilitators and barriers to participation (Shields et al., 2012). Personal barriers included lack of skill or coordination, preference for other activities, fear of injury, fear of being teased, not knowing what to do, self-consciousness, previous bad experiences, lack of time, and pain or discomfort. Social barriers included parental attitudes and behavior (e.g., lack of support, time, money, and opportunity), lack of friends, and negative attitudes of others. Environmental barriers included inadequate and inaccessible facilities and lack of transport. Policy and program barriers included lack of appropriate activities, lack of trained staff, negative attitudes of staff, and cost (Shields et al., 2012). While many of the facilitators and barriers were common to those identified in research involving children and young people without disabilities, for example, a child’s preference for the activity, cost, and time constraints, others were more clearly related to their disability; these included pain or discomfort, fear of incontinence, fear of being teased (Kang et al., 2007), negative perceptions of disability (from peers, staff, and others), and inadequate or inaccessible facilities and/or programs. The review did not examine, however, whether facilitators and barriers differed between young people engaged in OPA and those who did not participate or examine whether the type of disability and level of support needs influenced participation in OPA, as has been documented in prior studies (Mâsse et al., 2012; Darcy et al., 2016).

This is the first study, to our knowledge, that compared facilitators and barriers to OPA in two groups of young people with disability: those who currently participated in OPA and those who did not participate. This is an important distinction as, arguably, barriers and facilitators endorsed more frequently by non-participants would be an important focus for interventions to improve participation. This study also focused explicitly on OPA, rather than the broader topic of PA, due to research indicating that participation rates are lower for OPA than for PA for young people with disability (Solish et al., 2010; Arim et al., 2012). Situating the study within a PYD framework, this study addressed two research questions: (1) Do parents of young people with disability who do not participate in OPA differ in the facilitators and barriers they perceive compared to those who do participate in OPA? (2) Do young people with disability who do not participate in OPA differ in the type of individual assets (disability type, level of support needed, strengths, regular PA) and external assets (supportive relationships, communities, opportunities, financial resources) to those who do participate in OPA?

Potential facilitators were drawn from the fPRC, namely, the within-person factors (preferences, activity competence, and sense of self) hypothesized to be associated with participation and the young person’s involvement (motivation, persistence, social connection, happiness) during OPA. Potential barriers were identified from a review of the OPA literature (King et al., 2009; Shields et al., 2012). Participation was hypothesized to be positively associated with OPA that was experienced as intrinsically rewarding and offered opportunities to learn and acquire life skills such as persistence (internal assets) and to be positively associated with young people who had supportive relationships, resources, and opportunities (external assets). Supportive relationships were operationalized in this study as sibling and parental involvement in OPA, and positive coaching style. Resources and opportunities were measured by household income, access to OPA (distance, cost, time, environment), and National Disability Insurance Scheme (NDIS) support. In Australia, individuals with permanent and significant disability can apply to be supported by the NDIS which provides funding for supports and services. Families in this study were asked whether their young person was supported by the NDIS which was posited as an external asset as these supports can be used to assist with daily living, to participate in community activities, to increase independence, and to pursue goals. Other potential barriers and facilitators were measured by comparing participants and non-participants on demographic factors, parent-reported child strengths, and parent-reported amount and frequency of moderate and vigorous PA. In line with the abovementioned research questions, this study aimed to (1) compare facilitators and barriers to OPA for young people with disabilities who participated and those who did not participate and (2) utilize a PYD framework to assess whether the groups differed in the type of internal and external assets they reported.

## Materials and Methods

### Participants

The sample comprised 218 young people aged 4–17 years (mean age: 10.58) with a diverse representation of disabilities. They were categorized as being current OPA participants (*n* = 131) or not participating in OPA (*n* = 87) after completion of an online survey by parents or guardians. Surveys were completed by parents/guardians in this study to avoid over-burdening children and youth with the comprehensive list of barriers and facilitators we were interested in. Ninety-four percent of parent/guardians who responded were female (mean age: 42.9). When young people had more than one condition associated with disability, the condition with the greatest impact (as identified by the parent) was designated as the primary condition. Table 1 presents the frequency of primary disability categories, number of comorbidities, level of support, and their association with participation in OPA.

**TABLE 1:** Primary condition, comorbidities, level of support, and OPA participation.

Primary condition	Total frequency (%)	Current OPA *N* = 131 (60.09)	No OPA *N* = 87 (39.91)	*p*
ASD	89 (40.83)	51 (57.30)	38 (42.70)	0.381
Cerebral palsy	20 (8.77)	12 (60)	8 (40)	
Intellectual disability	19 (8.72)	13 (68.42)	6 (31.58)	
Down syndrome	14 (6.42)	9 (64.29)	5 (35.71)	
Depression/anxiety	15 (6.58)	6 (40)	9 (60)	
Attention deficit hyperactivity disorder	15 (6.58)	10 (66.67)	5 (33.33)	
Vision impairment	10 (4.59)	5 (50)	5 (50)	
Hearing impairment	8 (3.51)	8 (100)	0 (0)	
Rare genetic	7 (3.07)	4 (57.14)	3 (42.86)	
Diabetes	4 (1.75)	4 (100)	0 (0)	
Epilepsy	5 (2.19)	3 (60)	2 (40)	
Severe speech disorder	5 (2.19)	3 (60)	2 (40)	
Spina bifida	4 (1.75)	1 (25)	3 (75)	
Developmental coordination disorder	2 (0.88)	1 (50)	1(50)	
Other	1 (0.44)	1 (100)	0 (0)	
No. comorbidities				0.952
None	67 (30.73)	42 (62.69)	25 (37.31)	
One	46 (21.10)	28 (60.87)	18 (39.13)	
Two	42 (19.27)	23 (54.76)	19 (45.24)	
Three	30 (13.76)	19 (63.33)	11 (36.67)	
Four	18 (8.26)	11 (61.11)	7 (38.89)	
Five or more	15 (6.88)	8 (53.33)	7 (46.67)	
School support				0.308
None	58 (27.49)	31 (53.45)	27 (46.55)	
2–3 days per week	72 (34.12)	48 (66.67)	24 (33.33)	
3–5 days per week	81 (38.39)	49 (60.49)	32 (39.51)	
Ability to walk unassisted				0.253
Yes	201 (92.20)	123 (61.19)	78 (38.81)	
No	17 (7.80)	8 (47.06)	9 (52.94)	
Ability to understand simple instructions				0.003
Yes	202 (92.66)	127 (62.87)	75 (37.13)	
No	16 (7.34)	4 (25)	12 (75)	

### Procedure

The study was approved by the Deakin University Human Research Ethics Committee (2016-336). An Australia-wide purposive sampling strategy was conducted by advertising through the Australian National Disability Insurance Agency (NDIA) portal, the Australian Football League (AFL) and various sporting clubs, disability support organizations and Facebook pages. The advertising material consisted of a promotional flyer with a link to an online survey, Plain Language Statement, and consent information. Organizations were asked to promote the advertising material through their appropriate channels (e.g., websites and E-newsletters). Hardcopy advertising materials were also available for the organizations that wished to distribute them, for example, in clinic waiting rooms. Participants most frequently reported hearing about the survey via online social media (*n* = 91), followed by a disability support organization (*n* = 57). Fewer participants heard about the survey from “other” sources (*n* = 26), a sporting club (*n* = 14), word-of-mouth (*n* = 10), and the AFL (*n* = 4).

### Materials

The online survey was administered using the survey platform Qualtrics with items developed by a team of health professionals including pediatricians, psychologists, physiotherapists, sports scientists, and public health experts. It consisted of 99 items encompassing child and parent demographic questions, questions pertaining to the young person’s disability, current OPA participation, level of moderate and vigorous PA, and a list of facilitators and barriers to OPA (see Supplementary Materials for a copy of the survey).

Twelve potential barriers identified from a review of the OPA literature (King et al., 2009; Shields et al., 2012) were listed with a five-point Likert scale (strongly disagree, disagree, not sure, agree, strongly agree). They included individual barriers (child preferences, lack of skill, fear of injury, social difficulties), family barriers (time, cost), and environmental barriers (distance, cost, coaching style, unsuitable environment, activities too challenging, or not challenging enough). Items were recoded from “strongly disagree,” “disagree,” and “not sure” to a binary variable: 0 = no barrier. “Agree” and “strongly agree” were recoded to a binary variable: 1 = barrier.

Using the fPRC model of participation-related constructs, five factors hypothesized to contribute to the young person’s involvement in OPA (motivation, persistence, social connection, happiness, and involvement in the activity) were presented with a five-point Likert scale. Items were recoded from “does not describe my child” and “describes my child slightly well” to a binary variable: 0. Items were recoded from “describes my child moderately well,” “describes my child very well,” “describes my child extremely well” to a binary variable: 1. Three facilitators relating to the young persons’ preference for OPA (importance, meaningfulness, preference), two related to activity competence (improvement in skill and performance, increased level of independence performing the activity), and three facilitators relating to sense of self (confidence in ability to perform the activity, general self-confidence, and feelings of satisfaction and pride) were presented with a five-point Likert scale and recoded to a binary variable. If the young person was not currently involved in OPA, parents were asked to respond based on past involvement in OPA.

Parents were asked to report their child’s level of moderate and vigorous PA, any positive or negative experiences of OPA, and whether any siblings participated in OPA. The level of support needed by the young person was measured with three items (“does your child receive additional support in school,” “is your child able to walk without assistance,” and “is your child able to understand simple instructions”). Utilizing a strengths-based approach, parents were asked to list their child’s strengths which were then categorized using the Values in Action (VIA) classification of strengths (Wagner et al., 2019).

### Analysis Plan

The five-point Likert scale responses were recoded into binary variables. Although this method can diminish power, the relationship between the underlying construct (perception of barrier vs. non-barrier) and the dependent variable (OPA participation) was not necessarily linear; hence, a binary split was considered appropriate. Chi-square tests were conducted to explore if OPA participants and non-participants differed in their disability condition, number of comorbidities, the facilitators and barriers to OPA they endorsed, or their demographic characteristics. Factors were included in binary logistic regressions based on theory for facilitators (the inclusion of constructs from the fPRC model) and significance value for barriers (barriers that were significant at *p* < 0.05 in the chi-square analyses) to identify significant predictors of participation. Three separate binary logistic regressions were undertaken to identify significant barriers and facilitators (research question 1) and significant internal/external assets (research question 2).

## Results

Sixty percent of the sample (*n* = 131) were currently engaged in OPA which is less than the estimated 74% participation rate of OPA for all Australian children (Australian Sports Commission, 2018). The most common activities engaged in by participants in this study were swimming, soccer, dance, basketball, and gymnastics which is identical to the top six activities for all Australian children in 2017 (Australian Sports Commission, 2018). In this sample, 24% of the participants who currently engaged in OPA participated four or more times a week, 53% participated two to three times a week and 23% participated once a week. Parents were asked to report the amount of time their child spent in moderate and/or vigorous PA per week. This data was used to calculate whether the young people in the study were meeting the Australian government’s PA guidelines of 60 min of moderate to vigorous PA per day. Only 35% of the sample met this PA guideline which is similar to rates for Australian children in general (30% of children aged 2–17 met the PA guidelines; Australian Institute of Health and Welfare, 2018). Frequency of OPA participation was significantly associated with meeting PA guidelines [χ^2^(3) = 26.27, *p* = 0.000, Cramer’s V = 0.355]. Participants who engaged in OPA four or more times a week were almost 10 times more likely to be meeting PA guidelines compared to young people who did not participate at all, *b* = 2.29, *p* = 0.000, OR = 9.86, 95% CI [3.79, 25.64]. Participants who participated two to three times were three times more likely to be meeting PA guidelines than non-OPA participants, *b* = 1.11, *p* = 0.003, OR = 3.04, 95% CI [1.45, 6.34].

Although there was a wide representation of disabilities in the sample, 41% listed autism spectrum disorder (ASD) as their primary diagnosis. Sixty-nine percent of the sample had at least one comorbid condition, and 48% of the sample had two or more conditions. There was no significant difference in OPA participation between disability types or the number of comorbidities. Young people who could not understand simple instructions were significantly less likely to be OPA participants. Table 2 presents demographic information for young people with disabilities and their families. The only significant difference between those who participated in OPA and those who did not was household income and type of schooling. Those families in the lowest income bracket [χ^2^(2) = 6.80, *p* = 0.033, ϕ = 0.208] and young people not attending mainstream school were significantly less likely to be engaged in OPA [χ^2^(1) = 5.04, *p* = 0.025, ϕ = 0.149].

**TABLE 2:** Demographic characteristics of the sample.

		OPA (%)	Not in OPA (%)	*p*
		*N* = 131 (60.09)	*N* = 87 (39.91)	
Gender				0.055
	Male	75 (55.15)	61 (44.85)	
	Female	56 (68.29)	26 (31.71)	
Age in years				0.496
	4–8	38 (53.52)	33 (46.48)	
	9–11	40 (60.61)	26 (39.39)	
	12–14	27 (67.50)	13 (32.50)	
	15 +	26 (63.41)	15 (36.59)	
Indigenous status		4 (57.14)	3 (42.86)	0.838
Main language not English		0	3 (100)	0.059
Family type				0.581
	Single parent	18 (52.94)	16 (47.06)	
	Both parents	87 (62.59)	52 (37.41)	
	Other family	10 (58.82)	7 (41.18)	
	Missing	16 (57.14)	12 (42.86)	
Employment status of parent				0.081
	Full-time	31 (65.96)	16 (34.04)	
	Part-time	56 (63.64)	32 (36.36)	
	Home duties	20 (45.45)	24 (54.55)	
	Student/volunteer	8 (80)	2 (20)	
	Missing	16 (55.17)	13 (44.83)	
Household income				0.033*
	Below median HI	19 (46.34)	22 (53.66)	
	Middle median HI	39 (67.24)	19 (32.76)	
	Above median HI	41 (70.69)	17 (29.31)	
	Missing	32 (52.46)	29 (47.54)	
Parent education				0.819
	Year 10 or equivalent	14 (53.85)	12 (46.15)	
	Year 12 or equivalent	41 (61.19)	26 (38.81)	
	Certificate/diploma	35 (64.81)	19 (35.19)	
	Bachelor degree	25 (59.52)	17 (40.48)	
	Missing	16 (55.17)	13 (44.83)	
No. of siblings				0.470
	None	17 (54.84)	14 (45.16)	
	One	53 (67.09)	26 (32.91)	
	Two	29 (59.18)	20 (40.82)	
	Three +	16 (53.33)	14 (46.67)	
	Missing	16 (55.17)	13 (44.83)	
Education				0.036*
	Mainstream	103 (64.78)	56 (35.22)	
	Special	24 (51.06)	23 (48.94)	
	Other	4 (33.33)	8 (66.67)	
NDIS supported				0.814
	Yes	35 (61.40)	22 (38.60)	
	No	96 (59.63)	65 (40.37)	

* Indicates *p* < 0.05.

### Research Question 1: Do Parents of Young People With Disability Who Do Not Participate in OPA Differ in the Facilitators and Barriers They Perceive Compared to Those Who Do Participate in OPA?

Table 3 presents the percentage of barriers endorsed by parents of OPA participants and non-participants and chi-square analyses. Six barriers were endorsed significantly more by parents of children *not* currently participating in OPA. Using these six barriers as predictor variables in a binary logistic regression (see Table 4), the model explained 21% of the variance in OPA participation [χ^2^(6) = 26.39, *p* = 0.000, *R*^2^ = 0.21]. The only predictor that remained significant in the model was “there are no activities available that my child enjoys.” Young people of parents who endorsed this barrier were almost four times as likely to *not* be participating in OPA.

**TABLE 3:** Frequencies and Chi-square results for barriers to OPA.

Barriers	Current OPA		No OPA			
	*N*	%	*n*	%	χ^2^(1)	*p*
No activities my child enjoys	12	9.83	25	33.78	17.25	0.000
Too far to travel	34	27.87	32	43.24	4.86	0.027
Unsuitable/inconvenient time	26	23.21	26	36.62	3.84	0.050
Activities too costly	68	56.67	39	53.43	0.19	0.660
Do not have time to attend	26	22.03	11	15.49	1.21	0.272
Difficulty performing activities	58	52.25	43	74.14	7.59	0.006
Environment not suitable	27	24.32	23	40.35	4.63	0.031
Worries about being hurt/injured	24	20.17	24	32.88	3.90	0.048
Difficulty socially with peers	66	55.00	48	65.75	2.17	0.141
Activities too challenging	51	42.86	43	58.90	4.66	0.031
Activities not challenging enough	5	4.35	3	4.17	0.00	0.952
Coaching style not suitable	31	28.81	21	33.33	0.51	0.477

**TABLE 4:** Binary logistic regression for associations of barriers to OPA participation.

Barriers	*B*	*SE*	Significance	OR	95% CI
No activities my child enjoys	−1.39	0.46	0.002	0.25	0.10, 0.61
Too far to travel	−0.71	0.39	0.071	0.49	0.23, 1.06
Difficulty performing activities	−0.21	0.44	0.648	0.82	0.34, 1.97
Environment unsuitable	−0.51	0.42	0.231	0.60	0.26, 1.38
Worries about being hurt/injured	−0.23	0.44	0.609	0.80	0.33, 1.90
Finds activities too challenging	−0.45	0.42	0.287	0.64	0.28, 1.46
Constant	1.80	0.36	0.000	6.02	

Table 5 presents the percentage of facilitators endorsed by parents of OPA participants and non-participants and chi-square analyses. Using the facilitators in a binary logistic equation (see Table 6), the model explained 32% of the variance in OPA participation [χ^2^(13) = 38.84, *p* = 0.000, *R*^2^ = 0.32]. The only predictors that remained significant in the model were motivation and happiness. Young people of parents who reported that their child appeared unmotivated during OPA were 20 times more likely to *not* be participating in OPA and those that were unhappy during the activity were 12 times more likely to *not* be participating.

**TABLE 5:** Frequencies and Chi-square results for facilitators to OPA.

Facilitators	OPA		No OPA			
	*n*	%	*n*	%	χ^2^(1)	*p*
**Preference for OPA**						
Activity important to young person	100	81.30	28	63.64	5.65	0.017
Activity meaningful to young person	98	80.99	27	61.36	6.77	0.009
Preference for the activity	80	66.67	22	50.00	3.80	0.051
**Involvement in OPA**						
Appears motivated during the activity	108	87.80	21	47.73	29.62	0.000
Persists throughout the activity	96	78.05	24	53.81	7.86	0.005
Feels a social connection	89	72.36	21	48.84	7.89	0.005
Appears to be happy	106	86.18	32	74.42	3.14	0.076
Appears involved in the activity	105	85.37	27	61.36	11.27	0.001
**Activity competence/sense of self**						
Increase in skill and performance	110	90.16	31	70.45	9.82	0.002
Increased independence performing activity	113	91.87	29	65.91	17.16	0.000
Confidence in ability to perform activity	111	90.24	28	63.64	16.44	0.000
General self-confidence	105	85.37	29	65.91	7.74	0.005
Feelings of satisfaction and pride	108	87.81	31	70.45	6.99	0.008

**TABLE 6:** Binary logistic regression for associations of facilitators to OPA participation.

Facilitators	*B*	*SE*	Significance	OR	95% CI	χ^2^	Significance	*R*^2^
**Step 1 Preference for activity**								
Importance of activity	−0.59	0.89	0.508	0.56	0.10, 3.16	5.59	0.134	0.05
Meaningfulness of activity	0.85	0.82	0.298	2.35	0.47, 11.75			
Preference for activity	0.22	0.52	0.676	1.24	0.45, 3.44			
**Step 2 Involvement in OPA**								
Motivation during activity	3.03	0.86	0.000	20.59	3.84, 110.46	27.14	0.000	0.27
Persistence throughout activity	−0.92	0.77	0.233	0.40	0.09, 1.81			
Feels a social connection	0.42	0.53	0.420	1.53	0.56, 4.28			
Appears to be happy	−2.57	1.01	0.011	0.08	0.01, 0.56			
Appears involved in activity	0.83	0.92	0.365	2.30	0.38, 13.98			
**Step 3 Activity competence/sense of self**								
Increase in skill and performance	0.15	1.04	0.883	1.17	0.15, 8.98	6.12	0.295	0.32
Increased independence performing activity	1.06	1.17	0.365	2.88	0.29, 28.39			
Confidence in ability to perform activity	0.92	0.90	0.311	2.50	0.43, 14.64			
General self-confidence	−0.27	1.05	0.796	0.76	0.10, 5.98			
Feelings of satisfaction and pride	−0.67	1.07	0.532	0.51	0.06, 4.18			

### Research Question 2: Do Young People With Disability Who Do Not Participate in OPA Differ in the Type of Individual Assets (Disability Type, Level of Support Needed, Strengths, Regular PA) and External Assets (Supportive Relationships, Communities, Opportunities, Financial Resources) to Those Who Do Participate in OPA?

#### Individual Assets

Organized physical activity participation was not significantly associated with disability type, number of comorbidities, or ability to walk unassisted (see Table 1). Three individual factors were associated with OPA participation: ability to understand simple instructions, regular PA, and enjoyment of sport and/or PA. Young people who were not able to understand simple instructions were less likely to be OPA participants [χ^2^(1) = 8.87, *p* = 0.003, ϕ = 0.202]. Young people meeting recommendations of 60 min of moderate to vigorous PA per day were more likely to be OPA participants [χ^2^(1) = 18.02, *p* = 0.000, ϕ = 0.289] and young people who enjoyed sport and/or PA (listed as a strength by their parent) were more likely to be OPA participants [χ^2^(1) = 4.54, *p* = 0.033, ϕ = 0.144].

#### Supportive Relationships, Communities (External Assets)

Two factors were significantly associated with greater OPA participation: parent involvement in OPA (the parent volunteers as a coach) [χ^2^(1) = 4.59, *p* = 0.032, ϕ = 0.145] and having a sibling participating in OPA [χ^2^(1) = 12.57, *p* = 0.000, ϕ = 0.249]. Coaching style was not significantly associated with participation [χ^2^(1) = 0.51, *p* = 0.477].

#### Resources and Opportunities (External Assets)

Two factors differed significantly between OPA participants and non-participants. Those families in the lowest income bracket [χ^2^(2) = 6.80, *p* = 0.033, ϕ = 0.208] and young people not attending mainstream school were significantly less likely to be engaged in OPA [χ^2^(1) = 5.04, *p* = 0.025, ϕ = 0.149]. None of the other environmental factors examined in the study (cost, OPA environment and accessibility, distance to travel, coaching style, the competitiveness of other children and parents, NDIS support) were significantly different between the two groups. Using the three individual and four external assets in a binary logistic regression (Table 7), the young person’s love of sport, meeting PA recommendations and household income were significantly associated with current OPA participation [χ^2^(7) = 30.88, *p* = 0.000, *R*^2^ = 0.26].

**TABLE 7:** Binary logistic regression for associations of internal and external assets to OPA participation.

Internal and external assets	*B*	*SE*	Significance	OR	95% CI
Understands simple instructions	−0.87	0.91	0.339	0.42	0.07, 2.51
Meets PA recommendations	1.33	0.46	0.004	3.79	1.54, 9.33
Enjoys sport	1.31	0.58	0.023	3.71	1.20, 11.52
Parent participates in OPA	1.10	1.11	0.321	3.01	0.34, 26.65
Sibling participates in OPA	−0.44	0.43	0.305	0.64	0.28, 1.50
Household income	0.66	0.26	0.011	1.93	1.17, 3.19
School type	−0.46	0.34	0.171	0.63	0.32, 1.22
Constant	0.58	1.26	0.648	1.78	

## Discussion

This study aimed to compare facilitators and barriers to OPA for young people with disabilities who participated in OPA and those who did not participate and, utilizing a PYD framework, assess whether the groups differed in the type of internal and external assets they reported. Non-participation in OPA was significantly predicted by the barrier “there are no activities my child enjoys” and by a lack of motivation and happiness during OPA. Significant internal assets differentiating participants from non-participants were the ability to understand simple instructions, the parent-reported strength “love of sport/physical activity,” and meeting PA recommendations. Significant external assets were parent and sibling participation in OPA, school type (mainstream education), and household income.

In this study, motivation was the greatest predictor of participation. Parents who reported that their child was unmotivated when they participated in OPA (either currently or during past participation) were almost 20 times less likely to be currently participating in OPA. This finding accords with prior research findings that motivation is an important determinant of PA participation in both children and adults (Hurkmans et al., 2010; Pannekoek et al., 2013). For children, it is primarily intrinsic motivation, derived from the enjoyment of the PA itself, that is associated with participation in PA (Saebu and Sørensen, 2011; Sebire et al., 2013). This accords with the PYD position that activities need to be intrinsically rewarding if positive growth is to occur (Petitpas et al., 2005). Young people in this study who expressed happiness during OPA were 12 times more likely to be current OPA participants. Conversely, young people of parents who endorsed the barrier “there are no activities available that my child enjoys” were significantly less likely to be current participants. This is consistent with research indicating that continuous participation in OPA was contingent upon the enjoyment of the activity in studies of young people with disabilities (Heah et al., 2007; Nyquist et al., 2016) and those without disability (Garn and Cothran, 2006).

The importance of supportive relationships, resources, communities, and opportunities for positive youth development through OPA (Benson et al., 2007) was assessed in this study by examining the environment in which the activity occurs (suitability, distance to travel, level of competitiveness), the relationships (parental involvement, coaching style, peer interactions, sibling participation), and resources (household income, cost, availability of suitable programs). The only factors that differed significantly between participants and non-participants were school type, sibling and parent involvement in OPA, and household income. Families in the lowest-income bracket were five times more likely to be non-participants in this study, which suggests that costs associated with OPA were a significant barrier. In a large Australia-wide study of children’s participation in OPA led by the Australian Sports Commission, only 58% of children from low-income families participated in OPA compared to 84% from high-income families (AusPlay; Australian Sports Commission, 2018). Similarly, international studies have found that young people from lower socioeconomic status (SES) households are engaged in less OPA programs (Sallis et al., 1996; Kantomaa et al., 2007; Brockman et al., 2009). Interestingly, there is evidence to suggest that SES can also influence the type of support that parents provide to facilitate PA. Although this was not examined in the present study, previous research has found that higher SES families were more likely to enroll their children in a variety of OPA and co-participate in activities, whereas lower SES families were more likely to offer verbal encouragement and have children engaged in unstructured activities including outdoor play (Brockman et al., 2009; Noonan et al., 2017). While cost is a barrier that affects families with and without a child with disability, the cost of participation may be particularly onerous for families caring for a child with a disability due to the additional costs associated with disability care (therapies, equipment, loss of earnings due to parental care commitments) (Shields and Synnot, 2016).

Supportive relationships were assessed by examining parental involvement, coaching style, peer interactions, and sibling participation. The only factors associated with participation were sibling and parental involvement in OPA. Parents are recognized as one of the most important influences of PA in their children (Beets et al., 2010; Edwardson and Gorely, 2010; Smith et al., 2010), and many studies attest to the important role parents play in providing access, encouragement, and modeling active lifestyles (Beets et al., 2010). Children are more likely to be physically active when their parents are physically active, include the children in their activities, and provide encouragement and support (Davison et al., 2006). In this study, young people who had parents who volunteered as coaches were five times more likely to be participating in OPA; a similar finding to the AusPlay study (Australian Sports Commission, 2018), in which 75% of children who had at least one parent participating in OPA were OPA participants compared to only 56% of children who did not have a parent engaged in OPA.

The importance of sibling participation has previously been examined in a systematic review which found that siblings can facilitate engagement in OPA by acting as role models, offering encouragement and support, and enabling vicarious learning experiences (Blazo and Smith, 2018). In a study examining constraints to sports participation for people with disability, Darcy et al. (2016) found that a lack of friends or companions to participate with and not wanting to participate alone significantly hindered participation. In the current study, young people who had a sibling participate in OPA were three times as likely to be current OPA participants. The presence of a familiar sibling may encourage participation by providing emotional and practical support.

The other external factor that was significantly associated with participation in the present study was school type. Students enrolled in mainstream schooling were more likely to be OPA participants than students enrolled in special schools or special developmental schools. This finding may reflect the influence of more severe disability in students attending non-mainstream schools as young people with higher support needs were found to face greater constraints to OPA participation (Mâsse et al., 2012; Darcy et al., 2016). Similarly, in this study, higher support needs, measured by the ability to understand simple instructions, were significantly associated with participation. Young people who could understand simple instructions were five times more likely to be OPA participants. In a Canadian study of participation in young people aged 5–14 (*N* = 145,180) with neurodevelopmental disorders and disabilities and chronic medical conditions, severity of disability was the most important child characteristic to hinder participation (Mâsse et al., 2012). Although no significant difference in participation according to disability type was found in this study, attending non-mainstream schooling and not being able to understand simple instructions were significantly associated with non-participation, suggesting that these young people may have greater support needs which act as a barrier to participation. Future studies examining the impact of support needs on participation in OPA are warranted.

The only internal assets that differed between current OPA participants and non-participants were the parent-reported strength of love of PA/sport and meeting PA recommendations. It is unsurprising that youth who love PA and/or sport are more likely to be participants given the previous finding that enjoyment is a key driver of participation in young people. What remains to be answered is how to cultivate this love of PA in young people with disability. As previously discussed, the influence of family (parents and siblings) in modeling active lifestyles, facilitating access to OPA, and offering encouragement and praise is invaluable. Additionally, it is important to foster feelings of competence (self-efficacy) which has consistently been found to be a determinant of PA participation (Heah et al., 2007; Bauman et al., 2012). In this study, self-efficacy was measured using the fPRC items relating to increased skill, independence, and confidence in performing the activity. After including items relating to involvement (motivation, happiness, social connection, persistence) and items relating to preference (importance, meaning), self-efficacy was no longer a significant predictor of participation. Nevertheless, young people of parents who endorsed the barriers “my child has difficulty performing the activities” and “my child finds the activities too challenging” were significantly less likely to be participating in OPA, indicating that self-efficacy is an important contributor to participation. Perceptions of self-efficacy may be particularly significant to young people with disability as parents have noted the frustration and loss of confidence their children felt when they compared their skill level with other participants without disability (Shields and Synnot, 2016). The benefits of participating in adapted physical activities where skills can be developed in a safe and supportive environment were highlighted in a recent study (Nyquist et al., 2019). Children reported feeling comfortable learning new skills with other children with disabilities because they did not feel singled out or different. They also appreciated having sufficient time to develop mastery and felt optimistic that these newly acquired skills could be transferable to a mainstream OPA setting (Nyquist et al., 2019). Similarly, Shields and Synnot (2016) reported the need for inclusive pathways where children can progress from segregated activities through to mainstream or competitive sport.

Cultivating a love of PA in young people with disability by providing more supportive and enjoyable activities is an important way to assist youth to meet the Australian government’s recommendation of 60 min of moderate to vigorous PA per day. Only 35% of the participants in this study met these guidelines which is similar to rates for Australian children in general (30% of children aged 2–17 met the PA guidelines according to the Australian Institute of Health and Welfare, 2018); however, it has been suggested that there is an “amplified” concern of inactivity for young people with disability due to an increased risk of obesity, social isolation, and mental health concerns (Anderson and Heyne, 2010). Young people in this study who were not currently participating in OPA were significantly less likely to be meeting these PA recommendations; therefore, finding ways to increase OPA participation by addressing the facilitators and barriers identified in this study could assist in meeting the recommended daily PA. In a recent Australian study of PA levels during OPA, participants typically spent 40–50% of a sport’s practice session (e.g., soccer or netball) in moderate to vigorous PA as measured using an accelerometer (Ridley et al., 2018). The median duration of an OPA session was 1 h (Australian Sports Commission, 2018); therefore, one session would contribute to almost half the daily PA recommendation. In this study, 24% of the participants currently engaged in OPA participated four or more times a week, 53% participated two to three times a week, and 23% participated once a week. Current participation in OPA at these levels would not be sufficient to meet daily PA guidelines, and only 46% of the OPA group were meeting PA guidelines. Participation in OPA four or more times a week had the greatest odds of meeting PA guidelines; however, the cost to families engaging in OPA this frequently may be prohibitive.

There are a number of limitations in this study. Firstly, the sample included only three young people (1%) whose main language spoken at home was not English. This is significantly less than the 21% of Australians who speak a language other than English at home (Australian Bureau of Statistics, 2016). Seven young people (3%) were of Aboriginal or Torres Strait Islander heritage. This figure is representative of the Australian population (in 2016, Aboriginal and Torres Strait Islander people comprised 3.3% of the population according to the Australian Institute of Health and Welfare, 2019); however, compared to non-Indigenous Australians, Indigenous Australians are 1.8 times as likely to have a disability (Australian Institute of Health and Welfare, 2019). Future studies would benefit from ensuring the greater inclusion of young people whose main language at home is not English and Indigenous Australians. A further limitation relating to the sampling strategy was the use of predominantly online recruitment and an online survey. Online social media was the most frequently reported method of participants hearing about the study; however, families who have regular access to online material may not be representative of all families who have a child with a disability.

Additionally, the online survey was not previously trialed in the disability population, although survey items were derived from established models of participation (e.g., the fPRC), from a review of the OPA literature, and by consultation with a multidisciplinary team of health professionals. A power calculation was also not conducted due to limited research from which to estimate likely effect sizes. Instead, the sample size was based on pragmatic considerations, namely, the amount of data that could be collected without a significant increase in resources. Furthermore, during analysis, the five-point Likert scale responses were recoded into binary variables. Although this method can diminish power, many of the key relationships were significant; hence, if the five-point scale had been maintained, the relationships would be more likely to be significant. Consequently, this limitation does not compromise our confidence in the key conclusions.

Moreover, while the sample included a diverse range of disabilities, 41% of parents reported the young person’s primary disability to be ASD, consistent with data from the NDIS indicating that children on the autism spectrum currently comprise the largest primary disability category in Australia (National Disability Insurance Agency, 2018). Although there was no significant difference in OPA participation according to disability type, the over-representation of participants on the autism spectrum may have bearing on the types of facilitators and barriers that were endorsed as well as the internal and external assets reported. An additional limitation was the reliance on parent-reported facilitators and barriers. While we decided to collect information regarding barriers and facilitators to OPA engagement from the parent perspective to avoid over-burdening youth with the comprehensive list of barriers and facilitators we wished to investigate, other research involving young people with disability has successfully engaged young people in identifying facilitators and barriers (Heah et al., 2007; Shields and Synnot, 2016). Therefore, future studies comparing OPA participants and non-participants might benefit from including child-reported factors.

## Conclusion

This study confirmed prior literature in reporting that young people with disability do not participate in OPA at the same rate as their peers without disability. This is concerning given the weight of evidence which supports the potential for OPA to improve physical and mental health and to foster positive youth development (Murphy and Carbone, 2008; Holt et al., 2017). What this study adds to the literature is the identification of several factors that differentiate OPA participants from non-participants. Interventions to promote participation in OPA for young people with disabilities should firstly focus on ways to increase intrinsic motivation during OPA. Secondly, the experience of enjoyment is crucial for ongoing participation in OPA (Martin, 2006; Heah et al., 2007; Nyquist et al., 2016); therefore, interventions should focus on making OPA enjoyable. Thirdly, young people benefit when their family are also engaged in OPA. Interventions that promote participation of siblings and parents will facilitate participation of young people with disability. Finally, some young people are being hindered from participating due to a lack of financial resources. Supportive government policies to cover costs associated with OPA would lessen the financial burden on lower income families.

## Data Availability Statement

The datasets presented in this article are not readily available due to ethical considerations.

## Ethics Statement

The studies involving human participants were reviewed and approved by Deakin University Human Research Ethics Committee 2016: 336. Written informed consent to participate in this study was provided online by the participants, and where necessary, the participants’ legal guardian/next of kin.

## Author Contributions

NP and CE were involved in recruitment and data collection. MW analyzed the data. All authors participated in the conception and design of the study, were involved in data interpretation and manuscript drafting, and read and approved the final manuscript.

## Conflict of Interest

The Deakin Child Study Centre (NR and NP) receives philanthropic funding from Moose Toys, Ferrero Group Australia as part of its Kinder Joy of Moving pillar of Corporate Social Responsibility initiatives, MECCA Brands, Wenig Family, Geelong Community Foundation, and Grace & Emilio Foundation and industry partner funding from the Victorian Department of Education, to conduct research in the field of neurodevelopmental disorders and inclusion. The Deakin Child Study Centre (NR and NP) has also previously received scholarship funding from the Australian Football League and industry partner funding from the NDIS. NR has received donations from Vic Health and Bus Association Victoria; is a previous speaker honorarium from Novartis (2002), Pfizer (2006), and Nutricia (2007); and is a Director of the Amaze Board (Autism Victoria). None of the companies, industry partners, or organizational bodies listed above had a role in this research including the collection, analysis, and interpretation of data; in writing of the manuscript; and/or in the decision to submit the article for publication. The remaining authors declare that the research was conducted in the absence of any commercial or financial relationships that could be construed as a potential conflict of interest.
