# Supplementary material for: An Examination of Parent-Reported Facilitators and Barriers to Organized Physical Activity Engagement for Youth With Neurodevelopmental Disorders, Physical, and Medical Conditions
Source: Front Psychol. 2020 Sep 29;11:568723. doi: 10.3389/fpsyg.2020.568723 (PMC7550411; doi:10.3389/fpsyg.2020.568723)
Supplement: Supplementary file 1 [file Table_1.docx]

Supplementary Material

**Copy of Survey Questions**

What is your current involvement in organised sport and physical activity? Tick all that apply and specify what organisations you are involved in and the nature of your involvement:

- I am a Parent/carer of a child/children currently enrolled in organised sport/physical activity
- I am a Parent/carer of a child/children NOT currently enrolled in organised sport/physical activity
- I am a Parent/carer of a child/children NOT currently enrolled in organised sport/physical activity but would like to be
- I am a volunteer coach
- I am a paid coach
- I am both a paid coach and a volunteer coach

Please specify what sporting or physical activity organisations you are involved in as a parent, volunteer and/or staff member:

Following are some demographic questions. Hearing from people from all areas and backgrounds will support the development of appropriate services that can be provided to all areas. However, please skip any questions that you are not comfortable answering.

What is your gender?

- Male
- Female
- Other

What is your year of birth? (eg. 1965)

What state do you live in?

- Australian Capital Territory
- New South Wales
- Northern Territory
- Queensland
- Tasmania
- Victoria
- Western Australia
- South Australia

What is your postcode?

Are you of Aboriginal or Torres Strait Islander heritage?

- Yes, Aboriginal
- Yes, Torres Strait Islander
- Yes, both Aboriginal and Torres Strait Islander
- No

What is the primary language spoken at home?

- English
- Mandarin
- Cantonese
- Italian
- Greek
- Arabic
- Vietnamese
- Other ____________________

What is your highest level of qualification?

- Year 10 or equivalent
- Year 12 or equivalent
- Certificate/Diploma
- Bachelor degree
- Post-graduate degree

What is your current employment status?

- Home duties
- Employed part-time
- Employed full-time
- Employed casually
- Student
- Volunteer

How did you hear about this survey? Please tick all that apply.

- Disability support organisation
- Online social media
- Word of mouth
- AFL
- Sporting club
- Other (please specify) ____________________

How would you describe your family? Please choose all that apply.

- Single parent
- Sibling(s) take on additional responsibility to help with care
- Extended family, Aunts, Uncles and Grandparents are actively involved in supporting the children
- Step parents, parent(s)' partners are actively involved in supporting the children
- Both parents live together and are actively involved in supporting the children
- Parents do not live together but both take active roles in the children's lives
- Other (feel free to tell us more about your modern family) in the text box below ____________________

How many children do you have in total and what are their ages? (E.g. 3 children, aged 8, 10 and 12)

Do you have an example of a sport initiative, either in Australia or elsewhere, that encourages children with a range of disabilities or additional needs to participate in physical activity or sport?  Feel free to paste in links to news articles, videos, websites etc.

If yourself or your children are a member of a sporting club, please provide any feedback about your experience that you think would be relevant. In particular, how does the club accommodate your child's specific developmental or physical needs?

Tell us an example of when your child/children were supported well or when you or someone else (children or adults) supported someone in a great way.

The National Disability Insurance Scheme (NDIS) is the new way of providing support for Australians with disability, their families and carers. The NDIS provides two functions: individual plans for eligible people with a disability and Information, Linkages and Capacity Building (ILC) activities which will benefit all people with disabilities, their families and carers.  Are you aware of how the National Disability Insurance scheme could help you and your child?

- Yes. Please specify what you understand the NDIS to be and how it relates to your child. ____________________
- No
- Unsure. Please specify what you understand the NDIS to be and how it relates to your child. ____________________

What is your household income? We are asking this question to ensure we have collected information from people who are representative of Australia.   You may skip this item if you wish to.

- $0-$19,500
- $19,501 - $31,980
- $31,981 - $43,836
- $43,837 - $58,188
- $58,189 - $105,924
- $105,925 +
- Prefer not to respond

How many children do you have between 4 and 17 years of age who have a disability, or require extra planning and/or support to participate in physical activity? The term disability in this study refers to any condition that affects a child’s cognitive, developmental, sensory or mobility functions. If you have more than one such child, a specific response profile will be presented for each child. For example, if you have 3 children with a disability, the series of questions will be repeated 3 times in a loop.

- 1
- 2
- 3
- 4
- 5
- 6
- 7
- 8
- 9
- 10

Following is the series of questions for you to answer about your children. If you have more than one child with a disability, you will notice that these questions repeat. Please answer all questions with one of your children in mind, and if you see this message again, answer the questions with another one of your children in mind.

What is your child's gender?

- Male
- Female
- Other

When is your child's birthday? (dd/mm/yyyy)

What is your child's height? (in cm)

What is your child's weight? (in kg)

What kind of school does your child attend?

- Autism school
- Government school
- Catholic school
- Home school
- Independent/private school
- Special Developmental school
- Special school
- Specialist school
- Not in school
- Other (please specify) ____________________

Does your child receive additional support in school (personalised curriculum, teaching assistant or integration aide)?

- No, my child does not receive any additional support
- Yes, my child receives a half a day or less of additional support (support less than 2.5 days per week)
- Yes, my child receives more than a half a day of support (support more than 3-5 days per week.)

Has your child ever been diagnosed with any medical, cognitive or developmental challenges or disabilities? Please choose as many as apply.

- No diagnosis but this child struggles academically at school
- No diagnosis but this child struggles socially at school
- No diagnosis but this child struggles to participate in sport and physical activity
- ADHD
- Autism / ASD / Aspergers Syndrome / PDD-NOS
- Allergies that may prevent participating in organised physical activities
- Asthma
- Acquired Brain Injury (ABI)
- Bone, joint or muscle problems
- Cerebral Palsy (you can enter their GMFCS level if you know it). ____________________
- Chronic fatigue
- Diabetes
- Depression or anxiety
- Dyslexia and/or Dyscalculia
- Down Syndrome
- Cystic fibrosis
- Epilepsy/seizure disorder
- Hearing problems
- Heart abnormalities or concerns (murmur, arythmia, abnormal heart rate)
- Intellectual Disability
- Language Disorder
- Neural Tube Defect (Spina Bifida)
- Obsessive Compulsive Disorder (OCD)
- Oppositional Defiance Disorder (ODD)
- Stomach or bowel issues
- Rare or unknown disorder
- Problems with eyes or seeing properly (including wearing glasses)
- Other / additional. Sorry we couldn't list all choices, please write in the text box below. ____________________

If your child has been diagnosed with more than one condition, which of these conditions has the biggest impact on their daily life?

We would like to know a little more about your child's challenges. Is your child able to walk without assistance?

- Yes
- No

Is your child able to understand simple instructions? (E.g. go over to the tree).

- Yes
- No

Please tell us about your child's strengths (e.g. loves interacting with other children).

Is your child currently supported by the National Disability Insurance Scheme (NDIS)?

- Yes
- No

For the next four questions, please refer to the following definitions: Moderate physical activity: activities such as walking the dog, gardening and golf that cause your child to become a little bit breathless. Vigorous physical activity: activities such as tennis, jogging and cycling that cause your child to become quite breathless.

In a typical week, how many times does your child usually engage in vigorous physical activity for at least 10 minutes continuously? Please write the number.

Please estimate the total amount of time that your child usually spends engaging in vigorous physical activity in a typical week.

- Hours ____________________
- Minutes ____________________

In a typical week, how many times does your child usually engage in moderate physical activity for at least 10 minutes continuously? Please write the number.

Please estimate the total amount of time that your child usually spends engaging in moderate physical activity in a typical week.

- Hours ____________________
- Minutes ____________________

Which statement best describes your child's past and current involvement in organised physical activity?

- My child currently participates in organised physical activity
- My child does not currently participate in organised physical activity but has in the past
- My child does not currently participate in organised physical activity and has never participated
- My child has not participated in organised physical activity before but would like to

If your child has siblings, do these siblings participate in organised physical activity regularly?

- Yes. (please specify what organised physical activities/sports) ____________________
- No

What organised physical activities does your child participate in or would like to participate in? Each sport corresponds to one row. Skip the rows for sports that your child has never played and does not wish to play.

|  | Activity | | | How much did your child enjoy this activity | | | | Do you pay out-of-pocket fees (e.g. sports fees, travelling) for the activities selected? | | If there are out-of-pocket fees, how much did they cost you? This is optional to report |
| --- | --- | --- | --- | --- | --- | --- | --- | --- | --- | --- |
|  | Is currently playing | Has played in the past | Has not played but would like to | Hated it | Enjoyed it moderately | Loved it | N/A | Yes | No | Approximate cost per year ($) |
| Australian Rules Football |  |  |  |  |  |  |  |  |  |  |
| Baseball |  |  |  |  |  |  |  |  |  |  |
| Basketball |  |  |  |  |  |  |  |  |  |  |
| Bowling (5/10 pin) |  |  |  |  |  |  |  |  |  |  |
| Cricket |  |  |  |  |  |  |  |  |  |  |
| Cycling |  |  |  |  |  |  |  |  |  |  |
| Dance |  |  |  |  |  |  |  |  |  |  |
| Gymnastics |  |  |  |  |  |  |  |  |  |  |
| Hockey |  |  |  |  |  |  |  |  |  |  |
| Lawn bowls |  |  |  |  |  |  |  |  |  |  |
| Martial Arts |  |  |  |  |  |  |  |  |  |  |
| Netball |  |  |  |  |  |  |  |  |  |  |
| Quidditch |  |  |  |  |  |  |  |  |  |  |
| Rollerblading |  |  |  |  |  |  |  |  |  |  |
| Rugby |  |  |  |  |  |  |  |  |  |  |
| Soccer |  |  |  |  |  |  |  |  |  |  |
| Surfing |  |  |  |  |  |  |  |  |  |  |
| Swimming |  |  |  |  |  |  |  |  |  |  |
| Tennis |  |  |  |  |  |  |  |  |  |  |
| T-Ball |  |  |  |  |  |  |  |  |  |  |
| Other |  |  |  |  |  |  |  |  |  |  |

Please specify the type of organisations that your child participates in organised physical activities through. E.g. school, sporting club etc.

How frequently does your child participate in organised physical activities?

- 4 or more times a week
- 2-3 times a week
- Once a week
- Once a fortnight
- Monthly
- Once every 2-3 months
- Once every 4-6 months
- Once a year
- Never

How would you rate your child's views on this organised physical activity? If your child is not currently involved in an activity but has in the past, respond to these questions based on their past involvement.

|  | Strongly disagree | Disagree | Neutral | Agree | Strongly agree |
| --- | --- | --- | --- | --- | --- |
| This activity is important to the child |  |  |  |  |  |
| This activity is meaningful to the child |  |  |  |  |  |
| The child prefers this activity to other organised physical activities |  |  |  |  |  |
| The child prefers this activity to other activities in general (not just physical activities) |  |  |  |  |  |

While playing or being involved in organised sport/physical activity, how would you rate your child in the following areas?

|  | Does not describe my child | Describes my child slightly well | Describes my child moderately well | Describes my child very well | Describes my child extremely well |
| --- | --- | --- | --- | --- | --- |
| Appears motivated |  |  |  |  |  |
| Persists throughout the activity |  |  |  |  |  |
| Feels a social connection |  |  |  |  |  |
| Appears to be happy |  |  |  |  |  |
| Appears involved in the activity |  |  |  |  |  |

How has playing organised sport/physical activity influenced the following areas?

|  | None at all | A little | A moderate amount | A lot | A great deal |
| --- | --- | --- | --- | --- | --- |
| Improvements in skill and performance of the physical activity |  |  |  |  |  |
| Increased level of independence in performing the activity |  |  |  |  |  |
| Confidence in their ability to perform the activity |  |  |  |  |  |
| General self-confidence |  |  |  |  |  |
| Feelings of satisfaction and pride |  |  |  |  |  |

If given the opportunity, do you think your child would prefer to play in a modified league, disability specific league, or an inclusive league? Please rate each type on a scale of 0 to 10, where 0 - wouldn't like it at all and 10 - would love it.

- ______ Modified league- different rules and could include a different location such as a basketball court for wheelchairs
- ______ Disability specific league- Players all have the same condition such as cerebral palsy, autism, or intellectual disability
- ______ Side by side league- players play at the same time adjacent to the mainstream league
- ______ Inclusive mainstream- Players play alongside their peers

If given a choice, in what other ways would your child like to participate in physical activity other than playing? (eg. umpiring. score-keeping)

Does your child currently attend organised physical activity programs and participate in different ways? E.g. umpiring etc.

- Yes (please describe) ____________________
- No

Below are some possible benefits of participating in organised physical activities. For each statement, choose a response that tells us whether this is something you would hope your child might receive from participating in an organised physical activity.

|  | Definitely not a goal | Probably not a goal | Might or might not be a goal | Probably a goal | Definitely a goal |
| --- | --- | --- | --- | --- | --- |
| Making friends |  |  |  |  |  |
| Better physical health |  |  |  |  |  |
| Enjoy some fresh air |  |  |  |  |  |
| Reducing screen time with TV, phone, tablet, computer etc. |  |  |  |  |  |
| Have more energy |  |  |  |  |  |
| Increased coordination |  |  |  |  |  |
| Increased family time |  |  |  |  |  |
| An activity the child can do with their parent on the weekend |  |  |  |  |  |
| Increased concentration |  |  |  |  |  |
| Increased confidence |  |  |  |  |  |
| Other: please feel free to tell us more |  |  |  |  |  |

Out of the statements above, what are the TOP THREE benefits you feel your child might receive from being involved in organised physical activity?

- Making friends
- Better physical health
- Enjoy some fresh air
- Reducing screen time with TV, phone tablet, computer etc
- Have more energy
- Increased coordination
- Increased family time
- An activity the child can do with their parent on weekends
- Increased concentration
- Increased confidence
- Other: please feel free to tell us more ____________________

There are some things that can make it challenging for children to participate in organised physical activities. Below are some reasons children may not choose to participate in these activities. For each reason, choose a response that tells us whether you agree that this is a barrier to participating in organised physical activities for your child.

|  | Strongly disagree | Disagree | Not sure | Agree | Strongly agree |
| --- | --- | --- | --- | --- | --- |
| There are no activities available that my child enjoys |  |  |  |  |  |
| We have to travel too far to get to an activity |  |  |  |  |  |
| Activities happen at an unsuitable or inconvenient time (eg. when my child is tired) Please specify. |  |  |  |  |  |
| The activities are too costly |  |  |  |  |  |
| We do not have the time to attend activities |  |  |  |  |  |
| My child has difficulty performing the tasks required in the activities. Please specify. |  |  |  |  |  |
| The environment where the activities happen are not suitable for my child. Please specify. |  |  |  |  |  |
| My child worries about hurting/injuring themselves during physical activities. |  |  |  |  |  |
| My child has difficulty socially with peers at the activities. |  |  |  |  |  |
| My child finds the activities too challenging |  |  |  |  |  |
| My child does not find the activities challenging enough |  |  |  |  |  |
| The coaching style is not suitable for my child. Please specify. |  |  |  |  |  |
| Other (please specify) |  |  |  |  |  |

What are the TOP THREE barriers you feel you, your family or your child may encounter to achieving the goals and benefits you've selected above?

- Accessibility
- Cost
- Distance
- Other parents being too competitive
- Other parents not being competitive enough
- Other children being too competitive
- Other children not being competitive enough
- The game moves too fast for my child
- The game doesn't move fast enough for my child
- The coach is expecting too much
- The coach is not expecting enough
- There are no barriers, everything is going great
- Other- please feel free to tell us more ____________________

For the barrier below (that was selected in the previous question), please indicate how much of a barrier this is for you on the following scale, where 0 represents 'hardly a barrier', and 10 represents 'completely stops your family or child achieving the benefits of organised physical activity'.

- ______ Accessibility

For the barrier below (that was selected in the previous question), please indicate how much of a barrier this is for you on the following scale, where 0 represents 'hardly a barrier', and 10 represents 'completely stops your family or child achieving the benefits of organised physical activity'.

- ______ Cost

For the barrier below (that was selected in the previous question), please indicate how much of a barrier this is for you on the following scale, where 0 represents 'hardly a barrier', and 10 represents 'completely stops your family or child achieving the benefits of organised physical activity'.

- ______ Distance

For the barrier below (that was selected in the previous question), please indicate how much of a barrier this is for you on the following scale, where 0 represents 'hardly a barrier', and 10 represents 'completely stops your family or child achieving the benefits of organised physical activity'.

- ______ Other parents being too competitive

For the barrier below (that was selected in the previous question), please indicate how much of a barrier this is for you on the following scale, where 0 represents 'hardly a barrier', and 10 represents 'completely stops your family or child achieving the benefits of organised physical activity'.

- ______ Other parents not being competitive enough

For the barrier below (that was selected in the previous question), please indicate how much of a barrier this is for you on the following scale, where 0 represents 'hardly a barrier', and 10 represents 'completely stops your family or child achieving the benefits of organised physical activity'.

- ______ Other children being too competitive

For the barrier below (that was selected in the previous question), please indicate how much of a barrier this is for you on the following scale, where 0 represents 'hardly a barrier', and 10 represents 'completely stops your family or child achieving the benefits of organised physical activity'.

- ______ Other children not being competitive enough

For the barrier below (that was selected in the previous question), please indicate how much of a barrier this is for you on the following scale, where 0 represents 'hardly a barrier', and 10 represents 'completely stops your family or child achieving the benefits of organised physical activity'.

- ______ The game moves too fast for my child

For the barrier below (that was selected in the previous question), please indicate how much of a barrier this is for you on the following scale, where 0 represents 'hardly a barrier', and 10 represents 'completely stops your family or child achieving the benefits of organised physical activity'.

- ______ The game doesn't move fast enough for my child

For the barrier below (that was selected in the previous question), please indicate how much of a barrier this is for you on the following scale, where 0 represents 'hardly a barrier', and 10 represents 'completely stops your family or child achieving the benefits of organised physical activity'.

- ______ The coach is expecting too much

For the barrier below (that was selected in the previous question), please indicate how much of a barrier this is for you on the following scale, where 0 represents 'hardly a barrier', and 10 represents 'completely stops your family or child achieving the benefits of organised physical activity'.

- ______ The coach is not expecting enough

For the barrier below (that was selected in the previous question), please indicate how much of a barrier this is for you on the following scale, where 0 represents 'hardly a barrier', and 10 represents 'completely stops your family or child achieving the benefits of organised physical activity'.

- ______ Other

What costs do you encounter that make it difficult for your child to engage in organised physical activity?

- Required equipment
- Sports fees
- Travelling costs
- Uniform
- Other. Please specify. ____________________

Are there any sports programs that you/your child do not participate in due to the cost of the program?

- Yes. Please specify the sport and how much it costs: ____________________
- No

If your child has additional needs (e.g. disability and health conditions) that require additional costs to participate in sports, what is your current arrangement? (eg. pay out of pocket, sponsored by organisations.)

What other arrangement (to remove the financial barriers) would encourage your child to participate in sports?

Would you feel comfortable for your child to wear something identifiable (e.g. a wrist band, sticker etc.) to assist sporting coaches in identifying children who may need extra support while engaging in organised physical activities?

- Yes. Please specify reason: ____________________
- No. Please specify reason: ____________________
